# Enhancing structural and functional properties of commercially available pea protein isolate for plant-based meat analogues using combined pH-Shift, high-intensity ultrasound, and heat treatments

**DOI:** 10.1016/j.ultsonch.2025.107342

**Published:** 2025-04-04

**Authors:** Assam Bin Tahir, Anees Ahmed Khalil, Hina Gull, Khubaib Ali, Najla AlMasoud, Taghrid S. Alomar, Abderrahmane Aït-Kaddour, Rana Muhammad Aadil

**Affiliations:** a University Institute of Food Science and Technology, Faculty of Allied Health Science, University of Lahore; b University Institute of Diet and Nutrition Sciences, Faculty of Allied Health Science, University of Lahore; c College of Food Science and Engineering, Yangzhou University, Yangzhou, Jiangsu 225127, China; d Department of Chemistry, College of Science, Princess Nourah bint Abdulrahman University, PO Box 84428, Riyadh 11671, Saudi Arabia; e Universit́ Clermont Auvergne, INRAE, VetAgro Sup, UMRF, 15000 Aurillac, France; f Department of Food Technology, Faculty of Agroindustrial Technology, University of Padjadjaran, Sumedang 45363 Jawa Barat, Indonesia; g National Institute of Food Science and Technology, University of Agriculture, Faisalabad 38000, Pakistan

**Keywords:** High-intensity ultrasound, Pea protein, Gelation, Solubility, Protein secondary structure

## Abstract

Diets based on pea protein have gained international recognition as a good substitute for meat or other main sources of protein. However, problems like gelling and emulsifying qualities make it difficult to use pea protein. To successfully overcome significant obstacles related to the use of pea protein in many industrial sectors, particularly meat, this study offers a combination of methods used to produce commercially accessible Pea Protein Isolate (PPI). High-intensity ultrasound (HIUS) at three magnitudes (2, 4, and 8 W/mL), heat at 60 °C, and pH at 10.0 were all integrated within the set. For artificial meat, PUHP_2_, PUHP_4_, and PUHP_8_ were the most promising of the nine treatments. After undergoing combined treatments (pH-shift, HIUS, and heat), favorable gelling was shown by treatments, emulsifying, and foaming properties while containing the ideal and desired protein size, as understood by the results in the gel electrophoresis. When treated PPIs were used to stabilize the sunflower oil-in-water emulsion, the emulsion capacity increased significantly for PUHP_2_, PUHP_4_, and PUHP_8_ (43.47 %, 46.57 %, and 40.90 % increase, respectively). Furthermore, solubility (for PUHP_2_, PUHP_4_, and PUHP_8_) had shown considerable (p < 0.05) improvement from 31.03 % ± 2.11 % (DPPI) to 53.33 % ± 2.3 %, 55.13 % ± 1.0 %, and 58.43 % ± 3.2 %, in SEM which accompanied by differences in the morphology of protein. This study’s gelling properties (2.512 ± 0.1 N, 2.604 ± 0.1 N, and 2.168 ± 0.3 N, for PUHP_2_, PUHP_4_, and PUHP_8_) were crucial, primarily from the standpoint of plant-based meat analogs. The processes proposed by this study pea protein will be enabled that has undergone this series of chemical and physical processes to proceed in the direction of far better meat substitutes. Overall, this research contributes to the advancement of pea protein’s use as an industrial protein and allows better usage of its hypoallergenic, non-GMO and high protein content.

## Graphical abstract

Modification of Pea Protein Isolate (PPI) by pH-shift, heat treatment and High-Intensity Ultrasound, along with structural and functional analyses of those modified PPI.

**Figure:**
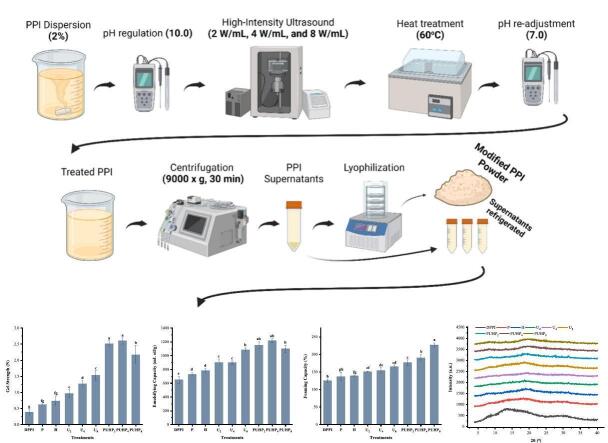

## 1 Introduction

Excessive use of animal products indicates wasteful use of valuable natural resources, especially when stable cereals are considered [1,2]. An increase of 70 % in food production appears to be essential given the high reach 9.7 billion growth of population is predicted by 2050. Pea protein (PP) has gained popularity and is attracting the interest of scientists worldwide due to its potential in beverage, meat, and other industries as a meat substitute, a component in meat analogs, and an ingredient in smoothies and mousses [3]. Globular proteins, which include parts of legumin, vicillins, and convicillins, make up approximately 65–80 % of PP [4]^.^ However, PP's industrial uses and functional properties are limited because of its distinctive spatial structures, which result in poor solubility [5].

There have been investigations on chemical, biological, and physical solutions to bridge the gap in terms of solubility and other functional properties [6]^,^ and pH-shift is a straightforward used method of changing the functional qualities of PP because studies have shown that heat and alkaline pH shift-reduce the size of the initially large faba bean protein aggregates, increasing their solubility and hydrophobicity [8]^.^ It has also been demonstrated that the PP structure unfolds with severe pH changes, producing a loose spatial confirmation, and then refolds at pH 7.0 [7].

One of the most reliable, efficient, quick, and accessible physical methods for causing partial unfolding of the PP structure is sonication. Acoustic waves of a specific frequency are used in this phenomenon, which is known as acoustic cavitation in protein studies [9]. Four main steps are involved in the process: rupturing, expansion, collision, and generation. Sonication combined with additional therapies had demonstrated significantly better outcomes in PP functionality [10]. The combined chemical and mechanical effects of ultrasound treatment can induce structural modifications at both the molecular and supramolecular levels of proteins [11,12]. This process leads to a reduction in molecular weight (MW), inhibits protein aggregation, and facilitates the exposure of previously buried hydrophobic groups, thereby enhancing their functional contributions [13]. These structural alterations may significantly improve the techno-functional properties of proteins, thereby expanding their potential for industrial applications. Furthermore, heat treatment is another widely employed approach for protein modification, influencing its structure and functionality.

In an attempt to push the limits of pea protein's industrial applications, current research focuses on the protein's functioning and applications. To improve the processing and functional qualities of plant proteins, several treatments have been investigated. Ultrasound and heat-assisted pH-shifting are two of these therapies. The combined impacts of heat, pH, and ultrasound to alter Functional properties and Pea Protein Isolate (PPI) structure, however, have not been thoroughly investigated.

This study used circular dichroism, XRD, and SEM to examine the effects of various combinations of modification treatments on morphology, crystallinity, and structural alterations. When paired with heat, pH, and ultrasound at magnitudes of 2 W/mL, 4 W/mL, and 8 W/mL, HIUS magnitude can result in conformational changes, which are considered necessary. Contrasted variations in functional properties such as solubility, the process of gelation the foaming process, and emulsifying were discussed.

## 2 Materials and methods

### 2.1 Materials

Pea Protein Isolate with protein concentrations of 80 %, 7 %, 5 %, 5 %, and 3 % for moisture, fat, total ash, and carbohydrate, respectively was procurred from Bacare & Co Ltd. (United Kingdom),. The Pierce Bradford Protein Assay Kit was purchased from Thermo Fisher Scientific Inc., and the SDS-Page Gel Rapid Processing equipment was provided by Sigma-Aldrich. Distilled water was utilized for the duration of the experiment, and all additional substances (not stated) were of analytical grade.

### 2.2 Characterization and preparation of the treated PPI

#### 2.2.1 PPI treatments and adjustments

Various adjustments, according to the following therapies: heat treatment, HIUS, and pH shift, were used to evaluate the impact on PPI. These combinations of modifications resulted in nine distinct treatments: DPPI (de-fatted pea protein isolate), P (pH-shifted pea protein isolate), H (Heat treated pea protein isolate), U_2_ (High intensity Ultrasound (2 W/mL) treated pea protein isolate), U_4_ (High intensity Ultrasound (4 W/mL) treated pea protein isolate), U_8_ (High intensity Ultrasound (8 W/mL) treated pea protein isolate), PUHP_2_ (pH-shifted high intensity Ultrasound (2 W/mL)-heat + pH-shifted pea protein isolate), PUHP_4_, (pH-shifted high intensity Ultrasound (4 W/mL)-heat + pH-shifted pea protein isolate) and PUHP_8_ (pH-shifted high intensity Ultrasound (8 W/mL)-heat + pH-shifted pea protein isolate). The parameters and adjustments made to each treatment are detailed in Table 1. Pea Protein Isolate (PPI) was defatted with n-hexane at a 1:10 (w/v) (PPI: n-hexane) ratio as part of the DPPI therapy. The two solutions were vigorously mixed for 90 min at room temperature, and after 30 min of rest, the n-hexane was decanted (this procedure was performed three times).

**Table 1:** Combinations and approaches used in PPI modification.

Sample	Defatting1:10 (w/v) (PPI:n-hexane)	Overnight Stirring (4 °C)	pH adjustment (10.0)	Sonication (W/mL, 15 min, <35 °C)	Heat treatment (60 °C, 15 min)	pH re-adjustment (7.0)	Reaction Time (min)
DPPI	√	√	×	×	×	×	×
P	√	√	√	×	×	√	60 min
H	√	√	×	×	√	×	15 min
U_2_	√	√	√	2 W/mL	√	√	90 min
U_4_	√	√	√	4 W/mL	√	√	90 min
U_8_	√	√	√	8 W/mL	√	√	90 min
PUHP_2_	√	√	√	2 W/mL	√	√	90 min
PUHP_4_	√	√	√	4 W/mL	√	√	90 min
PUHP_8_	√	√	√	8 W/mL	√	√	90 min

DPPI: De-fatted pea protein isolate, P: pH-shifted pea protein isolate, H: Heat treated pea protein isolate, U_2_: High intensity Ultrasound (2 W/mL) treated pea protein isolate, U_4_: High intensity Ultrasound (4 W/mL) treated pea protein isolate, U_8_: High intensity Ultrasound (6 W/mL) treated pea protein isolate, PUHP_2_: pH-shifted high intensity Ultrasound (2 W/mL)-heat + pH-shifted pea protein isolate, PUHP_4_: pH-shifted high intensity Ultrasound (4 W/mL)-heat + pH-shifted pea protein isolate, PUHP_8_: pH-shifted high intensity Ultrasound (8 W/mL)-heat + pH-shifted pea protein isolate.

To allow the remaining hexane to evaporate, the resultant PPI was let to rest for the whole night. To make a 2 % PPI dispersion, 5 g of defatted PPI (DPPI) was combined with 250 mL of distilled water. The dispersion was agitated for ten hours at 4 °C to get the best hydration. The same process used for PUHP_2_ was used to create the PPI dispersion. The dispersion was hydrated overnight using a pH meter (SevenExcellence, S400, Mettler-Toledo Philippines Inc.), allowed to come to room temperature (25 °C), and then promptly sonicated (2.0 W/mL, 15 min, maintained at < 35 °C) to control its pH (10.0) using a 2 M solution of sodium hydroxide (NaOH).

To prevent any accidental temperature changes, the produced dispersion was left to rest at room temperature (25 °C) for fifteen minutes. Following a 15-minute heat treatment at 60 °C, 2 M HCl was used to return the dispersion to pH 7.0. Using the proper HIUS and controlled heat or the pH level conditions for treatment, all subsequent treatments (P, H, U_2_, U_4_, U_8_, PUHP_4_, PUHP_8_) were carried out in accordance with the same protocols (Table 1). The supernatants were gathered after centrifugation (9000 × g, thirty minutes at 25 °C), and some of the results had been lyophilized for additional analysis and description.

#### 2.2.2 Solubility of proteins

With a few small modifications, the Bradford [14] method was used to evaluate the modified PPI's solubility. The Pierce Bradford Protein Assay Kit was used for this, and a standard curve was built using bovine serum albumin (BSA), a protein quantification standard. 250 μL of G250 Assay was added to the sample holes of a plate with 96 wells after a 5 μL treated protein sample had been obtained in triplicate. An ultraviolet (UV) spectrophotometer (SpectraMAX M2, Molecular Devices, Beijing, China) was used to detect the absorbance at a wavelength of 595 nm following ten minutes of incubation at room temperature (25 °C). Eq. (1) was used to determine the supernatant's protein concentration.

(1) $$ProteinSolubility\left(\%\right)=\frac{\mathrm{proteinconcentrationinsupernatant}}{\mathrm{initialproteinconcentration}}\times 100$$

#### 2.2.3 Size of protein Aggregate

The size of the particle analyzer (e Litesizer 500, Anton Paar, GmbH, Germany) was used to measure the particle size of aqueous solutions of PPI (4 mg/mL) were prepared. Every measurement was made three times at 25 °C.

#### 2.2.4 Turbidity

A UV/Vis spectrophotometer that was set to 600 nm in wavelength was used to measure the absorption of the PPI in the supernatants in order to quantify the turbidity, using distilled water as blank.

#### 2.2.5 Measurement of the circular dichroism (CD) spectrum

The spectrum of CD of a solution of PPI (0.1 mg/mL) which was in 10 mM PBS (pH 7.4) at 25 °C was assessed using the Chirascan V100, Applied Photophysics, UK. The optical path was determined at 0.1 cm using a quartz cuvette, the bandwidth was set at 1 nm, the scanning wavelength was maintained between 190 and 260 nm, and three scans were averaged. CDNN software was used to examine the data.

#### 2.2.6 **Surface hydrophobicity (**S_o_**)**

Kato and Nakai [15] state that in order to achieve a protein concentration of 0.01 mg/mL, the sample has been diluted with 0.1 mol/L PBS (pH 7.0). Four milliliters of each protein solution were then mixed with 40 Âµl of 1-anilino-8-naphthalene-sulfonate (ANS) tag solution and 8 mg per liter in 0.01 ml of PBS at pH 7.0 for each determination. After 40 s of shaking, the solutions were incubated in the dark for five minutes at 25 °C. The fluorescence spectrophotometer (FLUOROMAX-4, Horiba Instruments Inc. Edison, NJ, USA) with the fluorescence intensity (FI) at 390 nm (excitation) and 470 nm (emission) was used for the measurement with a fixed slit width of 5 nm. Plotting FI values versus protein concentration (represented by So) allowed for the establishment of the initial slope through the use of linear regression.

#### 2.2.7 Scanning electron microscopy (SEM)

Using a slightly altered version of protocol from Ali et al. [16], SEM images of the treated PPI were obtained using the JEOL-JSM 5910, (Japan) [16]^.^ The analysis was carried out at 14 kV and 1000 × after the samples were vacuum-sputter-coated with a gold–palladium combination. The PPI sample images were further examined through ImageJ software.

#### 2.2.8 Gel electrophoresis

With minor adjustments the methods of Zhi et al. [17], the SDS-Page was performed. Using a 12.5 % polyacrylamide gel in a 1.0 mm gel plate, 4 mg/mL with the distilled water samples were prepared, dissolved with buffer, and then centrifuged (12000 × g, 5 min, 25 °C). 18 μL of the prepared sample and 8 μL of the protein marker 10 k-250 k were placed onto precast gels for electrophoresis. The parameters were changed from 80 V for 10 min to 120 V for 60 min when the sample got closer to the separating gel. Following electrophoresis, the gel was stained with water, 0.1 % (w/v) Coomassie Brilliant Blue R-250 in 12 % (v/v) isopropanol, and 10 % (v/v) glacial acetic acid. The same solution was then used to de-stain the gel without the Coomassie Blue dye.

#### 2.2.9 Diffraction of X-rays (XRD)

Minor modifications were made to the XRD analysis, which was conducted in compliance with findings that had already been published by Ali et al. [18]. With tube voltage and tube current of 40 kV and 35 mA, respectively, the X'Pert Pro diffractometer (D6 PHASER, Bruker AXS Co., Ltd., Germany) was used to perform the XRD analysis. The following measurement settings were used: a scanning angle ranging from 4° to 40°; a step size of 0.02°; and a step size of 5 s.

#### 2.2.10 WHC (water holding capacity) and OHC (oil holding capacity)

Protocols explained by Ajibola et al. [19] were used to calculate WHC and OHC. 0.8 g of each sample was dissolved in 40 mL of distilled water (or oil) in 50 mL centrifuge tubes previously weighed to create samples with a concentration of 20 mg/mL. After a minute of vortexing and a half-hour of standing, the dispersion was centrifuged for 25 min at 25 °C at 7000 rpm. Excess water or oil was allowed to drip out for fifteen minutes before being disposed of after the supernatant was carefully gathered and decanted. Using the following formula, the quantity of water or oil retained per gram of sample was determined:

(2) $$\mathrm{WHC}/OHC\left(g\right)=\frac{W_{\mathrm{wetsediment}}-W_{\mathrm{drycomponent}}}{W_{\mathrm{drycomponent}}}$$

Where W_dry component_ is the dry protein powder's original weight prior to the addition of water or oil, and W_wet sediment_ is the final weight of sediment after water or oil has been obtained and decanted.

#### 2.2.11 Emulsion properties

*Preparation of emulsion*

A previously published study by Sha et al. [21] followed the procedure of making an oil-in-water emulsion with some slight changes. First, emulsification activity (EA) and capacity (EC) were evaluated by mixing 25 % (v/v) sunflower oil with 75 % (v/v) protein solution (10 mg/mL protein in 0.1 M phosphate buffer, pH 7.0). The mixture was homogenized at 13,500 RPM and in two 60-second place bursts utilizing a homogenizer (IKA T25 Digital Ultra. Turrax, IKA, Guangzhou, Guangdong, China). One-quarter of a part oil (v/v) was employed to generate the ultimate emulsion, which included 7.5 mg/mL of proteins.

Emulsifying activity index (EAI)

To create a combination of aliquots of 20 mL of produced emulsions, 20 mL of the emulsions were diluted 350 times and then dispersed in 7 mL of 0.1 % sodium dodecyl sulfate (SDS). The generated dilution's absorbance was measured at 500 nm, and its absorbance value was computed using Equation (4) to get the EAI.

(3) $$EAI\left(m^{2}/g\right)=\left[\left(4.606\right)/C*\left(1-\varphi \right)\right]*Abs*N$$

Emulsion capacity (EC)

Emulsion capacity was evaluated using the electric conductivity approach illustrated by Pearce et al. [22], and computational methods were performed by following the protocols of Cameron et al. [23]. Sunflower oil (400, 600, 800, 1000, 1200, and 1400 mL oil/g) was combined with a protein solution (10 mg/mL, pH 7.0) and homogenized (90 s, 13,500 RPM) using a homogenizer (IKA T25 Digital Ultra. Turrax, IKA, Guangzhou, China). Conductivity was measured using a MW301 PRO conductivity meter (Milwaukee Instruments, Inc., Rocky Mount, USA). EC was calculated using the quantity of oil (mL) electrically stabilized by 1 g protein, or mL oil/g.

#### 2.2.12 Properties of foaming

With a few minor modifications, foaming ability (FA) and stability (FS) were calculated as described by Xiong et al. [24]^.^ A 5 % PPI dispersion (20 mL) was made and whipped using a homogenizer (IKA T25 Digital Ultra. Turrax, IKA, Guangzhou, China) at 8000–10,000 RPM (with a 500 RPM increase every 15 s), for one minute, and at 25 °C. The foam was immediately prepared, and then thoroughly smoothed with a spatula after being put into a graduated cylinder. Once the volume of the foam was established, the foaming ability was calculated using Eq. (5).

(4) $$FA\left(\%\right)=\left(V_{o}/15\right)*100$$

where V_o_ is the volume of foam at two minutes.

After 15 and 30 min, the foam volume for FS was tested once more. Eq. (6) was used to calculate the values.

(5) $$FS\left(\%\right)={(V}_{f}/V_{o})*100$$

where, after 30 min, V_f_ is the final volume of the foam.

#### 2.2.13 Strength of gel

The gel preparation procedure by Chen and Campanella [25] was correctly followedwith a few little modifications. To create 200 mg/mL suspensions, pea protein samples were combined with 0.1 mol/l phosphate buffer solution (pH 7.4). The mixture was then vigorously shaken for an hour, allowed to de-aerate, and then put into glass vials with an inner diameter of 18.0 mm. After mixing, the suspension was heated at a rate of 1 °C per minute between 50 °C and 95 °C. After that, it was quickly chilled for half an hour in an ice slurry. Before analysis, the gels were incubated for one hour at room temperature (25 °C) following an overnight rest at 4 °C. A flat-ended stainless-steel probe (12.7 mm in diameter) was used to extrude the resultant gels at a crosshead speed of 1 mm/min until structural failure. The probe was connected to an analyzer for texture (TA. XT ExpressC, British SMS Co., UK). The gel strength (N) was the first piercing force needed to rupture the gels.

#### 2.2.14 Statistical analysis

A one-way analysis of variance (ANOVA) was used with a significance level of p < 0.05. The study was conducted using the Statistix 8.1 software (Analytical Software, St. Paul, USA). All sample measures were performed three times for accuracy, and the results were displayed as mean ± standard deviation (SD).

## 3 Results and discussion

### 3.1 Solubility

Pea protein's effectiveness in the sector is significantly influenced by its solubility. Table 2 shows that the treatments had a considerable impact on the samples' solubility. According to these findings, the patterns indicate that the sample's solubility elevated as the sonication intensity grew in tandem with the HIUS magnitude. Heat, pH, and different levels of HIUS were all responsible for changing the structure of the pea protein, which changed its size and allowed the structure to unfold partially [26]. Because of the combined impact of HIUS and other treatments, PUHP_8_ demonstrated the greatest solubility of all the treatments (58.43 % ± 3.2 %). Pea protein's increased solubility is crucial for its use in the beverage sector. The impacts of heat (40.07 % ± 5.0 %), pH (40.27 % ± 4.2 %), and HIUS (43.23 % ± 3.0 % for U_2_, 44.67 % ± 5.0 % for U_4_, and 48.70 % ± 3.3 % for U_8_) alone have also shown a significant (p < 0.05) influence on solubility, which was lesser than the combined treatment effects.

**Table 2:** Protein solubility, characterization, WHC, and OHC.

Sample	Solubility (%)	Turbidity (−)	Particle size (nm)	Surface Hydrophobicity (S_o_)	WHC (g/g)	OHC (g/g)	α-helix (%)	β-sheet (%)	β-turn (%)	Random coil (%)
							_______________Protein Secondary Structure__________			
DPPI	31.03 ± 2.1^e^	0.10 ± 0.0^d^	142.74 ± 2.5^a^	320.00 ± 8.6^f^	0.68 ± 0.0^a^	0.50 ± 0.1^e^	10.00 ± 0.4^ab^	34.60 ± 0.2^e^	22.50 ± 0.2^a^	32.10 ± 0.2^a^
P	40.27 ± 4.2^e^	0.25 ± 0.0^c^	136.80 ± 0.9^b^	331.67 ± 7.6^e^	0.27 ± 0.0^d^	0.51 ± 0.0^e^	10.40 ± 0.4^a^	37.80 ± 0.2^bc^	20.70 ± 0.2^c^	30.20 ± 0.2^c^
H	40.07 ± 5.0^d^	0.31 ± 0.0^b^	133.88 ± 1.7^b^	333.33 ± 2.8^e^	0.33 ± 0.0^d^	0.54 ± 0.0^de^	10.10 ± 0.3^ab^	38.10 ± 0.2^b^	20.90 ± 0.2^bc^	30.80 ± 0.2^b^
U_2_	43.23 ± 3.0^cd^	0.40 ± 0.0^a^	130.13 ± 1.3^c^	357.67 ± 3.0^d^	0.49 ± 0.1^c^	0.56 ± 0.0^cde^	10.10 ± 0.0^ab^	37.60 ± 0.2^c^	21.10 ± 0.2^b^	31.00 ± 0.2^b^
U_4_	44.67 ± 5.0^cd^	0.44 ± 0.0^a^	127.86 ± 0.6^cd^	365.00 ± 4.0^d^	0.53 ± 0.0^bc^	0.60 ± 0.0^cd^	10.40 ± 0.0^a^	48.10 ± 0.2^a^	17.00 ± 0.2^d^	29.20 ± 0.2^d^
U_8_	48.70 ± 3.3^bc^	0.43 ± 0.0^a^	125.83 ± 0.5^d^	389.33 ± 4.0^c^	0.58 ± 0.0^abc^	0.62 ± 0.0^bc^	9.80 ± 0.0^b^	48.10 ± 0.2^a^	17.00 ± 0.2^d^	29.20 ± 0.2^d^
PUHP_2_	53.33 ± 2.3^ab^	0.43 ± 0.0^a^	122.15 ± 3.3^e^	401.67 ± 7.5^b^	0.63 ± 0.0^ab^	0.68 ± 0.0^ab^	6.40 ± 0.3^c^	47.80 ± 0.2^ab^	17.10 ± 0.2^d^	29.30 ± 0.2^d^
PUHP_4_	55.13 ± 1.0^a^	0.42 ± 0.0^a^	117.27 ± 0.7^f^	412.33 ± 2.3^a^	0.67 ± 0.1^a^	0.71 ± 0.0^a^	6.40 ± 0.2^c^	45.67 ± 0.5^d^	21.20 ± 0.2^b^	31.80 ± 0.2^a^
PUHP_8_	58.43 ± 3.2^a^	0.41 ± 0.0^a^	104.67 ± 2.5^g^	416.67 ± 2.0^a^	0.59 ± 0.1^ab^	0.61 ± 0.0^bcd^	6.40 ± 0.1^c^	47.80 ± 0.2^ab^	17.00 ± 0.2^d^	29.30 ± 0.2^d^

DPPI: De-fatted pea protein isolate, P: pH-shifted pea protein isolate, H: Heat treated pea protein isolate, U_2_: High intensity Ultrasound (2 W/mL) treated pea protein isolate, U_4_: High intensity Ultrasound (4 W/mL) treated pea protein isolate, U_8_: High intensity Ultrasound (6 W/mL) treated pea protein isolate, PUHP_2_: pH-shifted high intensity Ultrasound (2 W/mL)-heat + pH-shifted pea protein isolate, PUHP_4_: pH-shifted high intensity Ultrasound (4 W/mL)-heat + pH-shifted pea protein isolate, PUHP_8_: pH-shifted high intensity Ultrasound (8 W/mL)-heat + pH-shifted pea protein isolate.

The reason for the change was that proteins were exposure to alkaline pH conditions (pH 10), which seemed to cause globulins to lose some sidechain connections while maintaining a relatively full structure, advocating for sample P (pH-shift alone) showed a smaller gain in solubility than other samples. The enhancement in the solubility could be attributed to the HIUS-induced acoustic cavitation, which disrupts the intra- and intermolecular interactions, leading to an increased exposure of charged groups. Furthermore, some researchers suggested that the primary mechanism for improved solubility involves the dissociation of native protein complexes into individual subunits. Additionally, conformational changes and the formation of new soluble aggregates may contribute to this effect. The protein solubility was significantly enhanced due to the structure unfold, which can be referred to as a “molten sphere”. In all individual HIUS (U_2_, U_4_ and U_8_) and combined PUHP treatments, ultrasound caused acoustic cavitation which increased the amount of charged groups and broke down certain intra- or intermolecular connections. Comparing P, H, U_2_, U_4_ and U_8_, with PUHP_2_, PUHP_4_, and PUHP_8_, indicates that pH-shift, heat treatment combined with HIUS significantly enhanced protein dissolution. Heat treatment played the role of cleaving hydrogen bonds between polypeptides, resulting in a reduction in molecular weight (MW) of pea protein isolates and increase in electrostatic repulsion. The main source of increased solubility was similarly found to be the disintegration of native protein complexes into their constituent subunits; conformational alterations and the generation of new soluble aggregates might be the secondary causes.

### 3.2 Turbidity and particle size

In Table 2, the outcomes of particle size, turbidity, and solubility are connected. Results regarding particle size were in line with the HIUS parameters that changed during the course of the treatments. Ultrasound and heat treatments were found to have an impact on pea protein particle size in earlier studies [27]. The present study has come to similar conclusions, demonstrating a substantial (p < 0.05) reduction in the size of the particle as the sonication intensity (W/mL) increased while the heat treatment (60 °C) for each treatment remained constant. PUHP_8_ had the lowest size of particle, measuring 104.67 nm ± 2.5 nm. By one of the previous findings, the turbidity of the DPPI sample was 0.10 ± 0.0 [28]. In comparison to the combined treatments, DPPI and P had reduced turbidity because the soluble portion of the protein had fewer particles, as indicated by the solubility data in Table 2. In contrast, Zhi et al. [17] showed that the combined treatments (PUHP_2_, PUHP_4_, and PUHP_8_) had the maximum turbidity because of the conspicuous particle abundance due to smaller particle size and thorough distribution in a suspension.

### 3.3 Circular dichroism (CD)

Table 2 displays the findings of an investigation of the changes in the protein's secondary structure using CD spectroscopy. The secondary structure of the protein was impacted by the processing conditions (pH-shift, HIUS, and heat) on PPI. A higher β-sheet and random coil can give the protein a complex secondary structure, which is typically one of the factors that results in better gelation [20]. Secondary structure is a crucial feature of pea proteins and can be responsible for functional characteristics like gelation and solubility.

PUHP_2_ showed significant changes in both β-sheet and random coil (13.2 % and 2.8 %, respectively), while pH-shift alone could only modify β-sheet by 3.2 % and random coil by 1.9 %. The trend exhibits a noticeable fluctuation when the magnitude of HIUS was raised in conjunction with heat and pH change, in contrast to DPPI. In the past, comparable outcomes were noted for rice protein [29]***.***

### 3.4 Surface hydrophobicity (S_o_)

Regarding the quantity of proteins' exposed hydrophobic residues of amino acids, S_o_ is thought to be a critical element influencing the gelation, foaming, and emulsifying qualities of proteins [30]. As shown in Table 2, the unmodified samples S_o_ had the lowest (320.00 ± 8.6^g^), while PUHP_8_ had the highest S_o_ (416.67 ± 2.0^a^). For P and H, their S_o_ values remained at a similar level, ranging from 331.67 ± 7.6^e^ to 333.33 ± 2.8^e^. Since S_o_ is a measure of the degree of contact between hydrophobic groups and water molecules, an increase in S_o_ may be the result of newly formed surfaces with various treatments. When protein molecules undergo a pH change, they may become loose and unfolded, exposing a number of hydrophobic residues on their surface [31]. As the pH shifted with sonication, it not only provided a powerful shearing force to break apart protein clumps but also released hydroxyl radicals that break down covalent bonds, lowering the molecular weight of proteins [32] and creating more new surfaces. Furthermore, heating may have caused further unfolding under pH-shifting circumstances, exposing more hydrophobic groups (in the case of PUHP_2_ to PUHP_8_). Likewise, Makinen et al. [33] discovered that after 5 min of heating at 100 °C and pH 10.5, the hydrophobicity of soy proteins rose by 50 %. Furthermore, compared to other approaches, the PUHP_8_′s higher S_o_ showed a more severe alteration for the PPI molecule. This might be because the pH-shifting, HIUS, and heat treatments were superimposed; nonetheless for PUHP_8_, the level of cleavage felt excessively harsh.

### 3.5 SEM

**Fig. 1:**
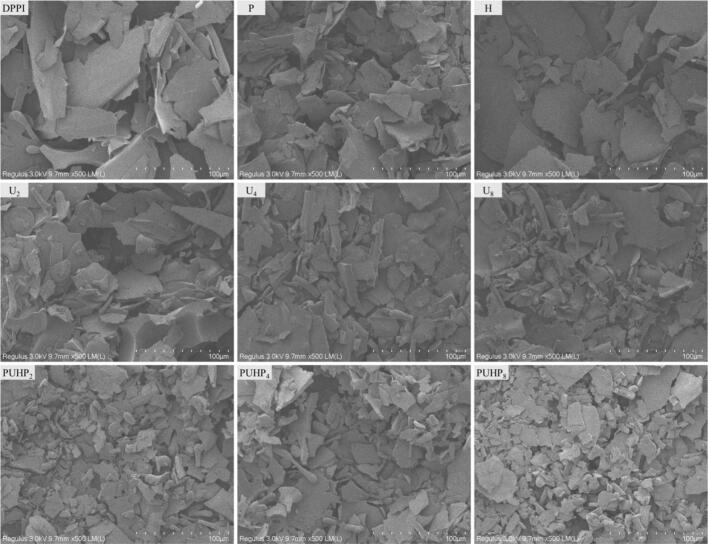
SEM micrographs of different freeze-dried PPIs, magnification was 500 × .

Protein morphology was examination displayed interesting results through SEM (Fig. 1). The morphologies seen in SEM pictures were significantly influenced by the treatments in comparison. When all of the treated PPI were compared, the DPPI exhibited protein aggregates; separated or powder-like forms were hardly observed, while a particle-based structure was revealed when the HIUS magnitude increased, as was evident in the earlier talks. The combination of heat (60 °C) and pH (10) may have contributed to the particle size reduction, the structures were impacted even though the pH and heat treatments were carried out under mild circumstances. As for HIUS, the acoustic cavitation during the sonication process was a definite cause of this alteration [10]. SEM figures demonstrated the differences brought about by individual (P, H, and U_2_–U_8_) and combination treatments. Since the morphologies varied, it may be concluded that the combination of treatments (heat, pH, and HIUS) were more successful than individual (P, H, U_2_, U_4_, and U_8_). Similar to other studies, several treatments also displayed holes in the structure that gave the impression of being nanofibers because of the combined processing of heat, HIUS, and pH-shift [34].

### 3.6 Electrophoresis in gel

**Fig. 2:**
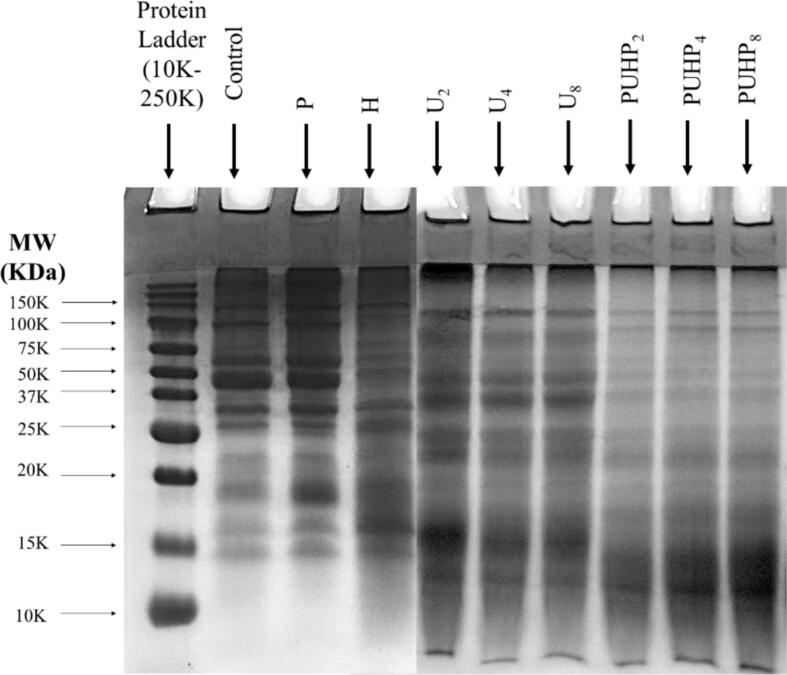
Reducing SDS patterns of DPPI and different treated PPIs.

The SDS-page findings allow for the visualization of PPI sub-units. The Winogradoff et al. [35] method was used to identify the band, and the changes in the proteins shown in Fig. 2 are depicted by the bands that formed in the gels. The hexameric (330–410 kDa) protein pea legumin (11S) is made up of basic polypeptides (19–22 kDa) and acidic polypeptides (38–40 kDa) that contains each subunit of approximately 60 kDa. Under reducing circumstances, sulfide bonds between polypeptides broke, separating the legume subunits into acidic (Lα) and basic (Lβ) polypeptides. While the bands at (12–16, 20, 25–36 kDa) were low Mw vicilin fragments, the bands around 90 kDa were formed by lipoxygenase. Because the HIUS parameters were changed based on the treatments, the bands were greatly impacted (Table 2). Only pH-shift treatment was applied to Sample P, hence the lack of sonication treatment in P caused the bands to move to lower Mw when compared to PUHP_2_, PUHP_4_, and PUHP_8_, highlighting the absence of physical alteration in the PPI. Since U_2_, U_4_ and U_8_, were only subjected to sonication (2, 4, and 8 W/mL), the change was minimal when compared to P and H, indicating the impact of HIUS magnitudes and amplifying the influence on the physical modification of PPI. Band alterations were quite evident for PUHP_2_, PUHP_4_, and PUHP_8_, and the results indicated that the combined treatments had led to a noticeable band shift toward the trails' lower MW side (11–20 kDa), where they were almost indistinguishable. The outcomes are consistent with those of the earlier studies [36,37,38]. The pattern of these findings indicates that combination treatments were crucial for PPI band placement, and the HIUS impact was evident when P, H, U_2_, U_4_, and U_8_ were compared. The structure and characteristics of PPI were significantly impacted by all of these treatments taken together, and they can change how well pea protein functions.

### 3.7 Water and oil holding capacity

WHC is the volume of water that one gram of the protein may retain in order to keep it from being expelled from the matrix [39]. OHC affects the mouthfeel and taste retention of food products, particularly meat, by indicating the amount of oil trapped in the protein matrix. The balance between particle size and protein molecular weight, protein conformation, and hydrophobicity seemed to be very important in terms of these properties. WHC (Table 2) was the highest for DPPI, which was not treated, along with PUHP_4_ with a negligible difference. WHC was the second-highest for PUHP_4_, indicating that this treatment has found the perfect balance to show superior qualities. For this sample to exhibit the second-highest WHC (0.63 ± 0.0^ab^), highest OHC (0.71 ± 0.0^a^), higher β-sheet, and random coil, a moderate pH-shift, heat, and HIUS magnitude supplied the balance. These structural and function-based characteristics all point towards a higher gel strength.

### 3.8 XRD

**Fig. 3:**
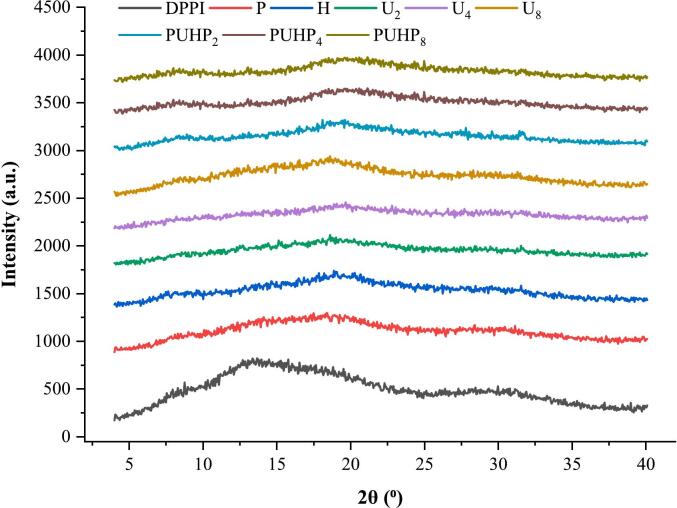
XRD patterns of all PPI treatments for the observation of crystallinity angle 4° − 40°.

XRD was used to evaluate the crystallinity of each PPI treatment; the findings are shown in Fig. 3. The XRD spectra of the treatments show broad peaks at between 10 and 22 (PUHP_2_, PUHP_4_, and PUHP_8_) indicated the existence of the polypeptide chain structure's α-helix and β-helix [40], but another investigation discovered that α-helix and β-helix were connected to peaks in soybean protein at 10 and 22 [41]. The sharper peak seen in the pattern of XRD of all treated proteins at around position 32 [42] was caused by the synthesis of NaCl, which was also seen in previous investigations. PPI had become more amorphous following the combination of treatments, as seen by the larger peaks in the XRD patterns as compared to the DPPI. Table 2 shows the decrease in crystallinity along with indicating an increase in protein solubility.

### 3.9 Emulsion properties

**Fig. 4:**
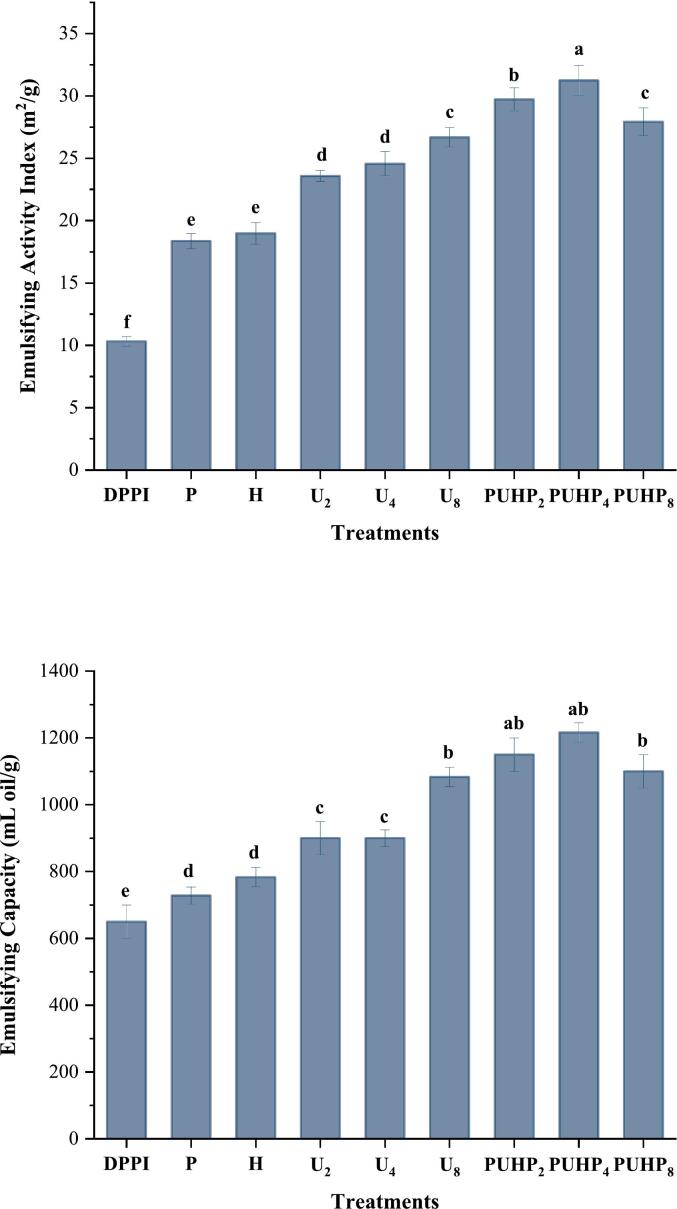
Emulsifying Activity Index (EAI) and Emulsifying Capacity (EC) of different PPIs relative to DPPI treatment.

The turbidity-based approach, in which Sha and Xiong [21] is a simple method of evaluating the interfacial modifications in protein emulsion properties, was used to identify emulsifying activity. The findings (Fig. 4) indicate a rising trend in the HIUS magnitude in conjunction with pH and heat. Previous studies have demonstrated similar outcomes with sonication [43,44], where ultrasound enhanced pea protein adsorption at the oil–water interface. Oil may attach to partly cleaved and changed proteins, which may explain why PUHP_4_ had the greatest emulsifying activity index (31.24 ± 1.1). In comparison to DPPI (650 ± 50), PUHP_4_ also had the highest emulsifying capacity (1216.67 ± 28.8) and P showed the lowest change in EAI (728 ± 25.6), both of which were significantly (p < 0.05) greater than DPPI. The findings established a comparable upward trend in emulsifying capabilities (apart from PUHP_8_) when the protein's hydrophobic layer (Fig. 4) was exposed and the particle size shrank, increasing the protein's surface area for oil binding. When comparing the emulsifying qualities of P, H, U_2_, U_4_, and U_8_, there was a visible difference, although not a remarkable one indicating a large impact. Nevertheless, they differed significantly from other combination therapies, which further demonstrates the impact of combined approaches.

### 3.10 Stability and foaming ability

**Fig. 5:**
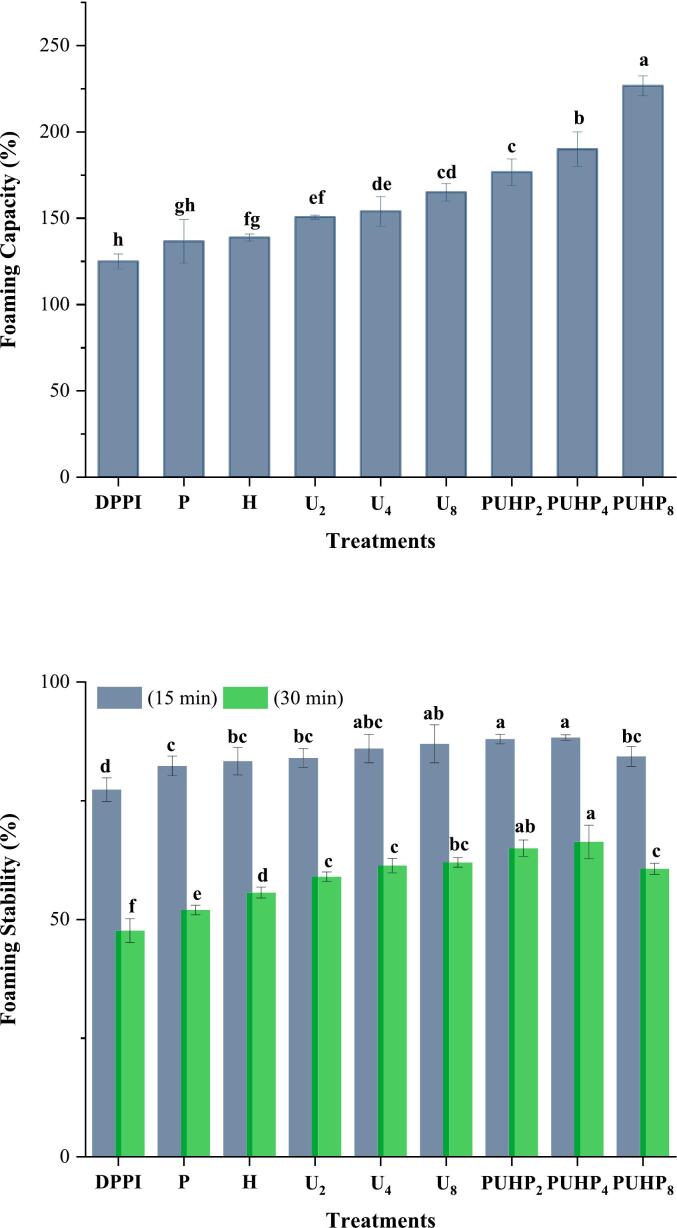
Foaming Capacity (FC%) of PPIs after freshly prepared foams & Foaming Stability (FS%) of PPIs at 15 and 30 min. Different superscript letters of the same index indicate significant difference (p < 0.05).

In Fig. 5, the foaming characteristics findings are displayed. Foaming stability was assessed for each treatment at two distinct time intervals (15 and 30 min). When compared to untreated PPI (DPPI), the foaming stability and capacity of treated PPIs exhibited an upward trend. Among all treatments, PUHP_8_ exhibited the highest foaming capacity (226.67 % ± 5.7 %) and the tiniest size of the protein. It is reasonable to assume that partial protein structure unfolding causes rapid adsorption at the newly formed air–water interface [45], which also resulted in better foaming stability of 84.33 % ± 2.0 % within the first 15 min. Comparing at 30 min, DPPI's stability dropped by 23.67 % and PUHP_8_′s by 16.67 %. PUHP_8_′s foaming stability declined much more quickly (around 24 %). Protein structure had an impact on foaming stability; a protein with a worse drainage system may have less foaming stability. In comparison to PUHP_2_ and PUHP_4_, this suggests that PUHP_8_′s secondary structure was somewhat less flexible due to the large magnitude of HIUS, which also suggests that its gelation properties are worse. Their (PUHP_2_ and PUHP_4_) enhanced solubility, foaming, hydrophobicity, and gelling may be due to a favorable balance they were able to establish between changing the structure and exposing the hydrophobic layer on the same time. Comparisons among individual treatments (P, H, U_2_, U_4_, or U_8_) and combined treatments (PUHP_2_, PUHP_4_, and PUHP_8_) revealed that pH, heat and HIUS all collectively were able to contribute more, resulting in the considerable (p < 0.05) increase in foaming characteristics. The degree of unfolding and protein modification to improve foaming properties was found to be significantly influenced by these procedures.

### 3.11 Gel strength

**Fig. 6:**
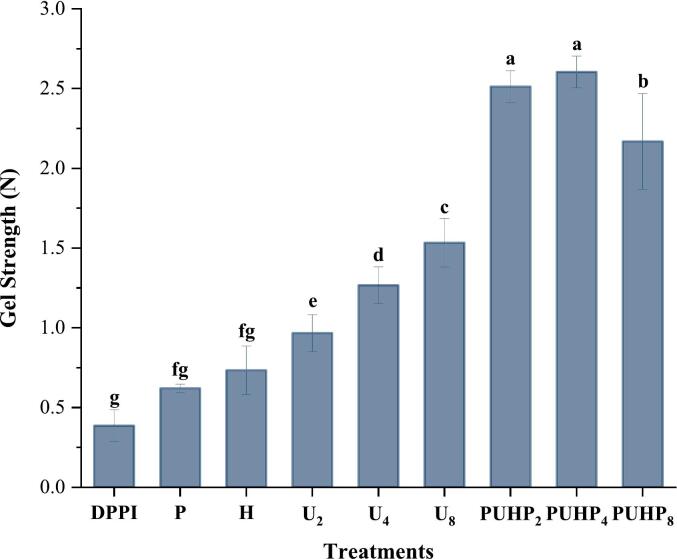
Gel strength of all PPI treatments for the observation of hardness of gels formed by 200 mg/mL PPI concentration. Different superscript letters indicate significant difference (p < 0.05).

All treatments showed that the gels created at 200 mg/mL were solid; tilting or shaking the gels did not cause any movement or alter their formation. Fig. 6 demonstrates that the weakest gels were produced by DPPI. Among the treated PPI, the weakest gels were formed for P and H (0.62 ± 0.0 N, 0.73 ± 0.1 N), and then it started to increase from U_2_ and continued to do so until PUHP_4_. The best gel structures for their industrial use were likely to be possessed by PUHP_2_ and PUHP_4_, although at PUHP_8_ due to the combination of techniques the gel strength was seen to be reduced. This might be attributed to vigorous processing which included a higher magnitude of sonication, this also points out a very clear and obvious effect on the structural and functional properties of HIUS magnitude. Nevertheless, the increased gel strength in the treatments where combined techniques were applied deemed suitable for industrial application [30,46]. In comparison to alternative therapies, the degree of cleavage in the previously listed procedures was perhaps optimal. Compared to PUHP_8_, where the PPIs had undergone a higher degree of breakage and alteration (due to higher HIUS magnitude), this degree of peptide chain breakage and molecule rupture may have caused the combination of polypeptides and amino acids to bind together and create a solid structure. pH-shift, heat treatment and HIUS, combined were responsible for achieving this ideal particle size, surface hydrophobicity and changes in the secondary structure. pH-shift was responsible for degradation of protein structure and disruption of disulfide bonds, heat treatment helped molecular weight distribution and formation of insoluble aggregates to enhance gelling, whereas HIUS helped with partial protein unfolding, modification in the secondary structure, enhancing protein-water interaction. Previously, various researches have been carried out to evaluate their effects on pea protein, this combination of techniques highlighted that optimal magnitudes and controlled extent of processing can result in an ideal protein, which was unachievable through individual treatments, as seen in the results.

## 4 Conclusion

Combining several methods, such as heat treatment, HIUS, and pH shift, has proven to be a successful way to alter PPI and change its secondary structure to suit industrial needs. Heat exposure, pH change, and HIUS combinations significantly changed the secondary structure of the protein. Because of the enhanced binding capacity, the combined treatments changed PPI's emulsification activity and prolonged the retention duration of foam droplets by exposing their hydrophobic and sulfhydryl groups. In comparison to the DPPI, individual treatments (P, H, U_2_, U_4_, and U_8_) displayed the results as the techniques were introduced, with HIUS having the highest effect on the structure and function of PPI due to the acoustic cavitation phenomena. The magnitude of HIUS proved its dominancy in the combined treatments as well, where gel strength started to reduce at PUHP_8_ due to the highest magnitude. Overall, in treatments PUHP_2_, PUHP_4_, and PUHP_8_, the study demonstrates that the combination procedures led to a notable enhancement of stability, emulsification ability and capacity, and dissolution. Especially remarkable were the positive gel strength results for the combined treatment samples, which demonstrated efficient effects of HIUS parameters paired with other techniques used to modify PPI. Once additional optimization is achieved, such alterations show potential for use in the derivation of meat alternatives/analogs.

## CRediT authorship contribution statement

**Assam Bin Tahir:** Conceptualization, Data curation, Methodology. **Anees Ahmed Khalil:** Conceptualization, Data curation, Writing – review & editing. **Hina Gull:** Writing – review & editing. **Khubaib Ali:** Writing – review & editing. **Najla AlMasoud:** Writing – review & editing. **Taghrid S. Alomar:** Writing – review & editing. **Abderrahmane Aït-Kaddour:** Writing – review & editing, Resources, Funding acquisition. **Rana Muhammad Aadil:** Writing – review & editing, Conceptualization.

## Declaration of competing interest

The authors declare that they have no known competing financial interests or personal relationships that could have appeared to influence the work reported in this paper.
